# Supplementary material for: An Intramolecular Hydroaminomethylation-Based Approach to Pyrrolizidine Alkaloids under Microwave-Assisted Heating
Source: Molecules. 2022 Jul 25;27(15):4762. doi: 10.3390/molecules27154762 (PMC9332719; doi:10.3390/molecules27154762)
Supplement: Supplementary file 1 [file molecules-27-04762-s001.zip › molecules-1795426-supplementary.pdf]

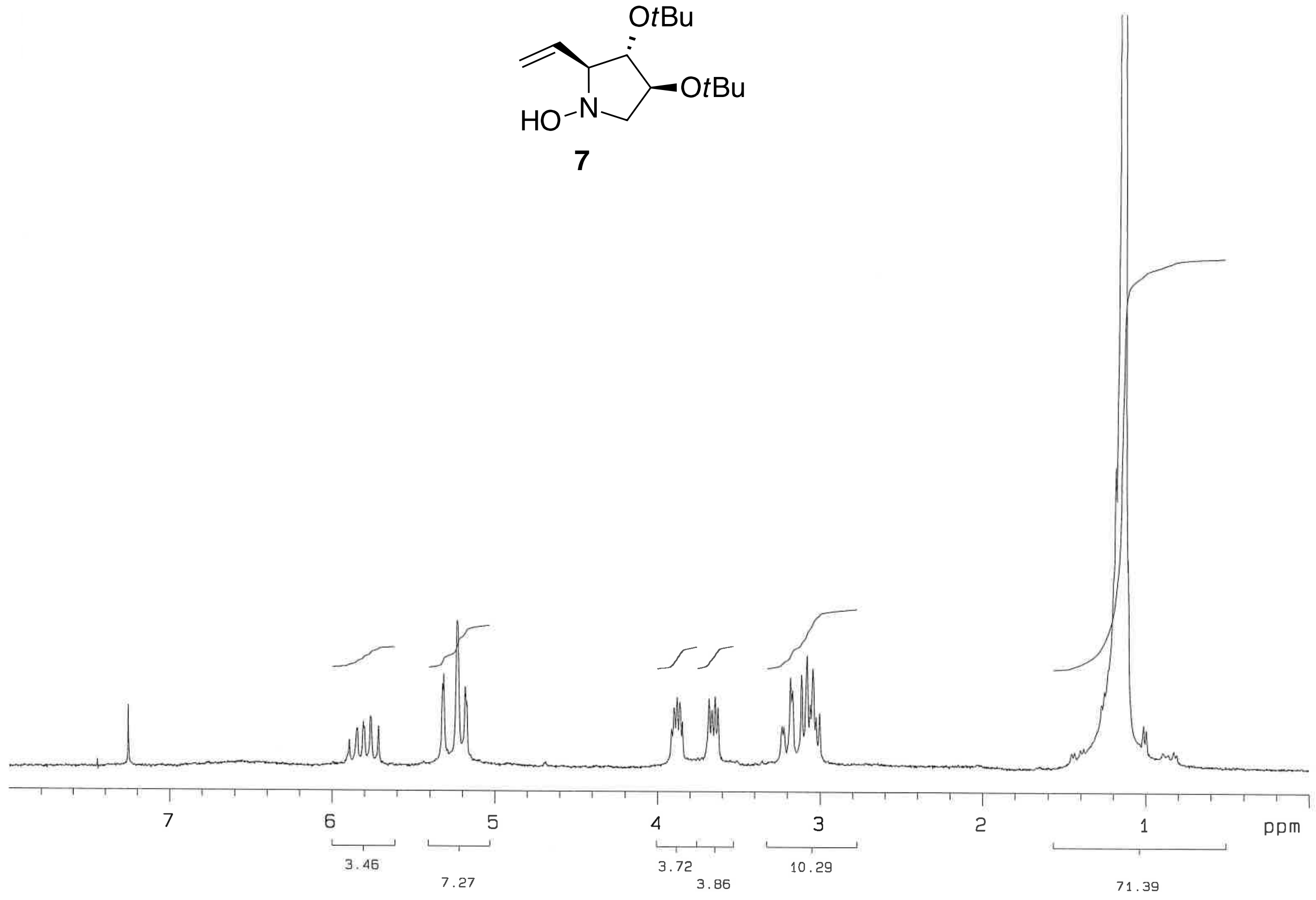

OtBu
OtBu
HO
N
7
7
6
5
4
3
2
1
ppm
3.46
7.27
3.72
3.86
10.29
71.39

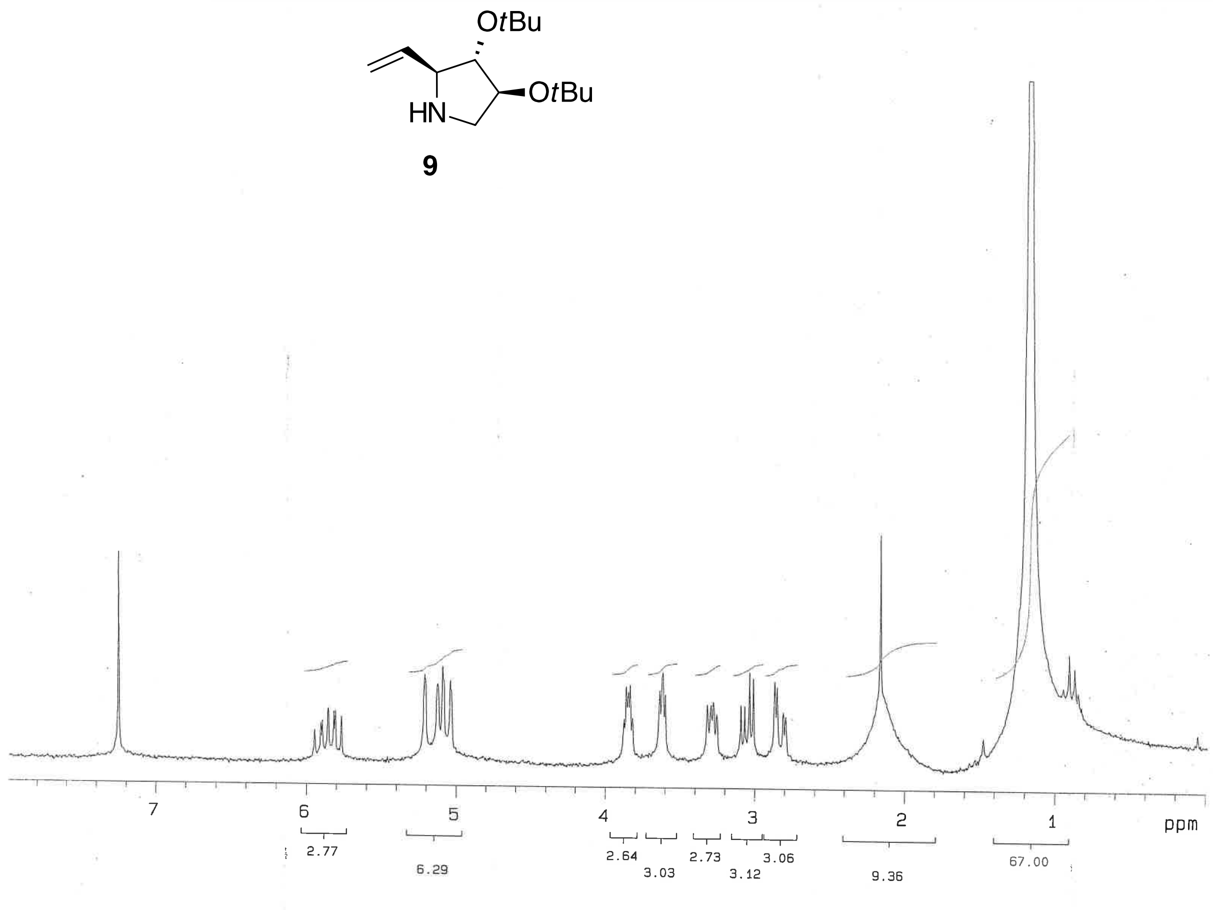

OtBu
OtBu
HN
9
7
6
5
4
3
2
1
ppm
2.77
6.29
2.64
3.03
2.73
3.12
3.06
9.36
67.00

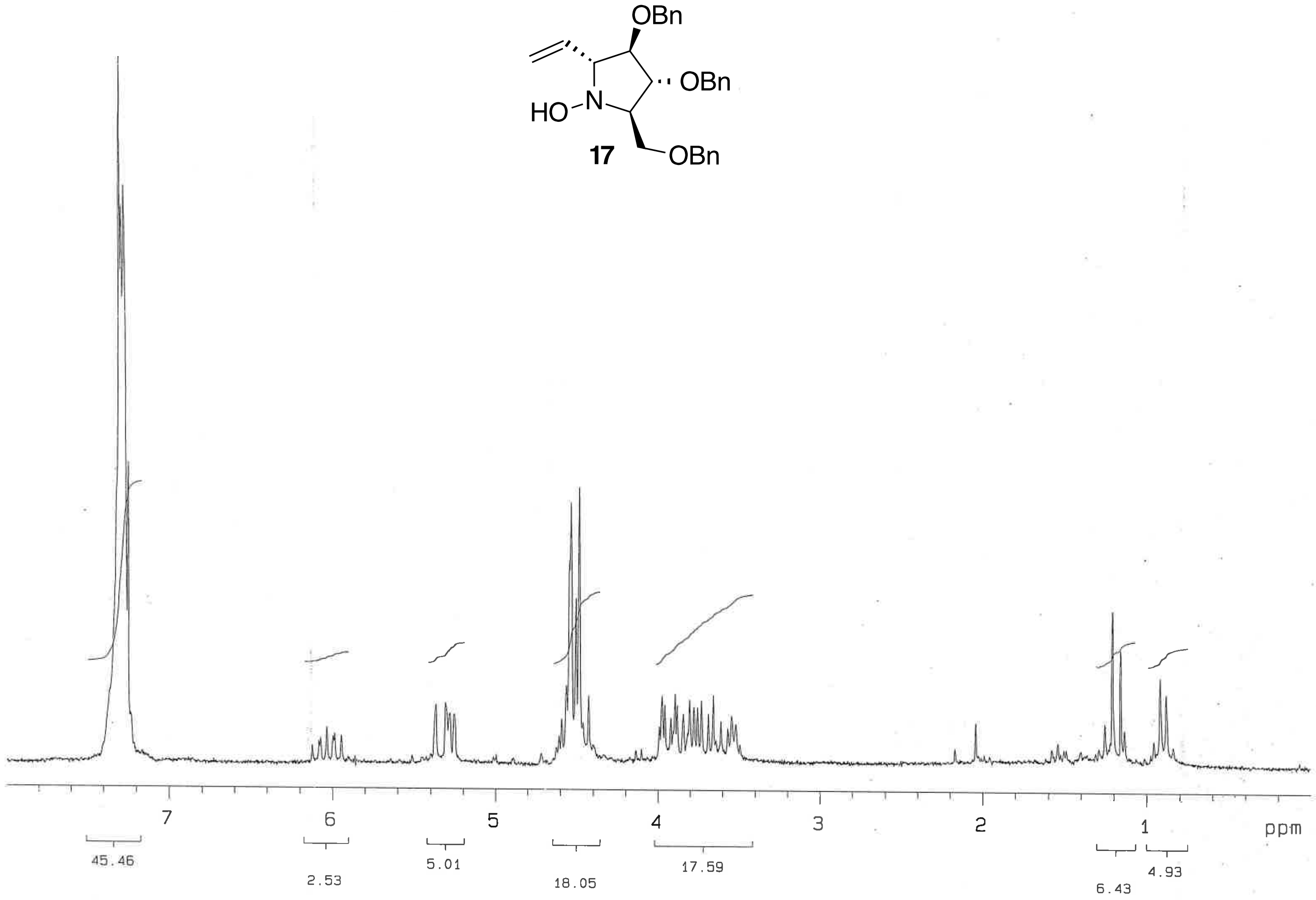
OBn
OBn
HO
N
OBn
7
6
5
4
3
2
1
ppm
45.46
2.53
5.01
18.05
17.59
6.43
4.93

17

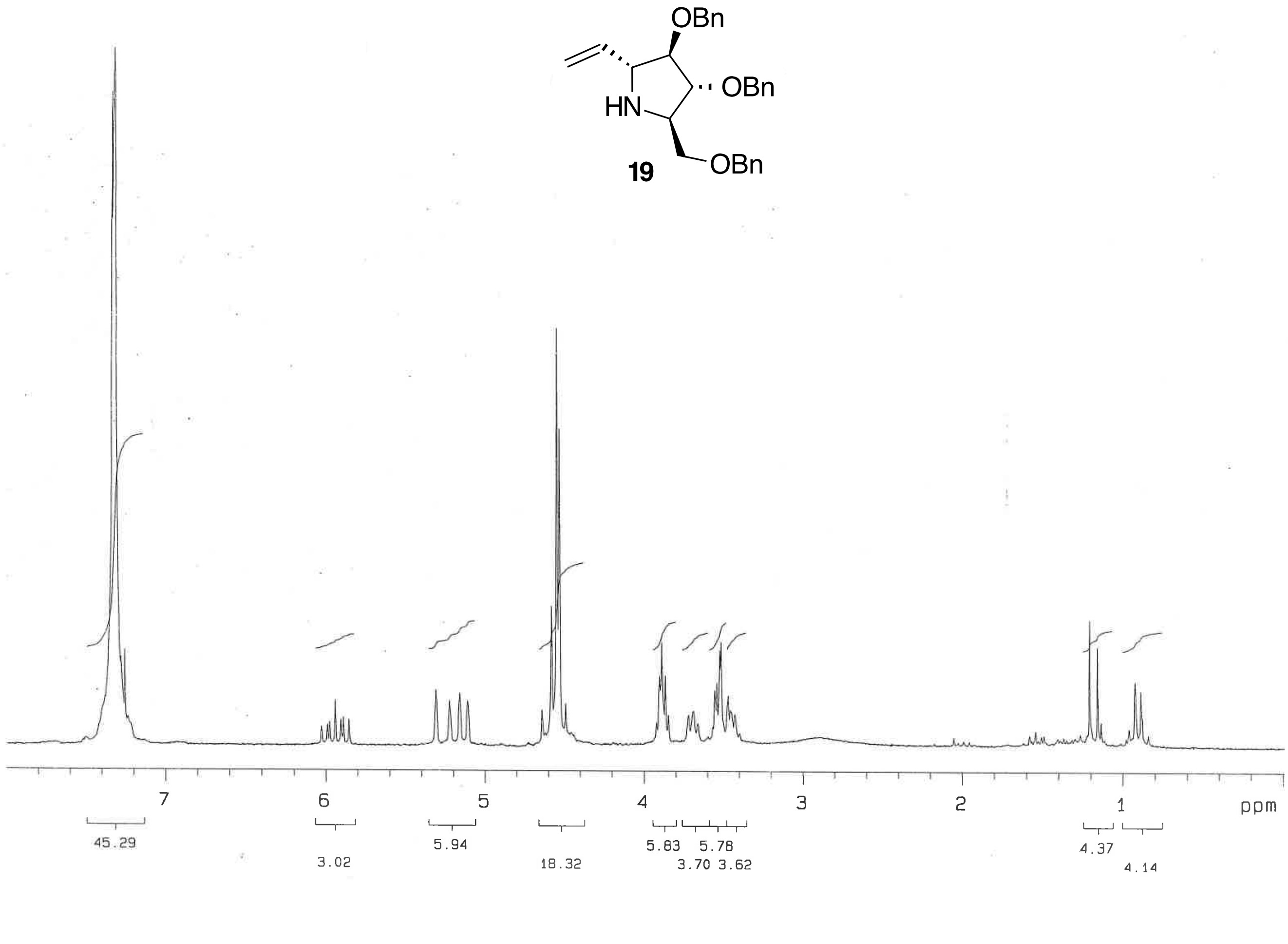
OBn
OBn
HN
OBn
7
6
5
4
3
2
1
ppm
45.29
3.02
5.94
18.32
5.83
3.70
5.78
3.62
4.37
4.14

19

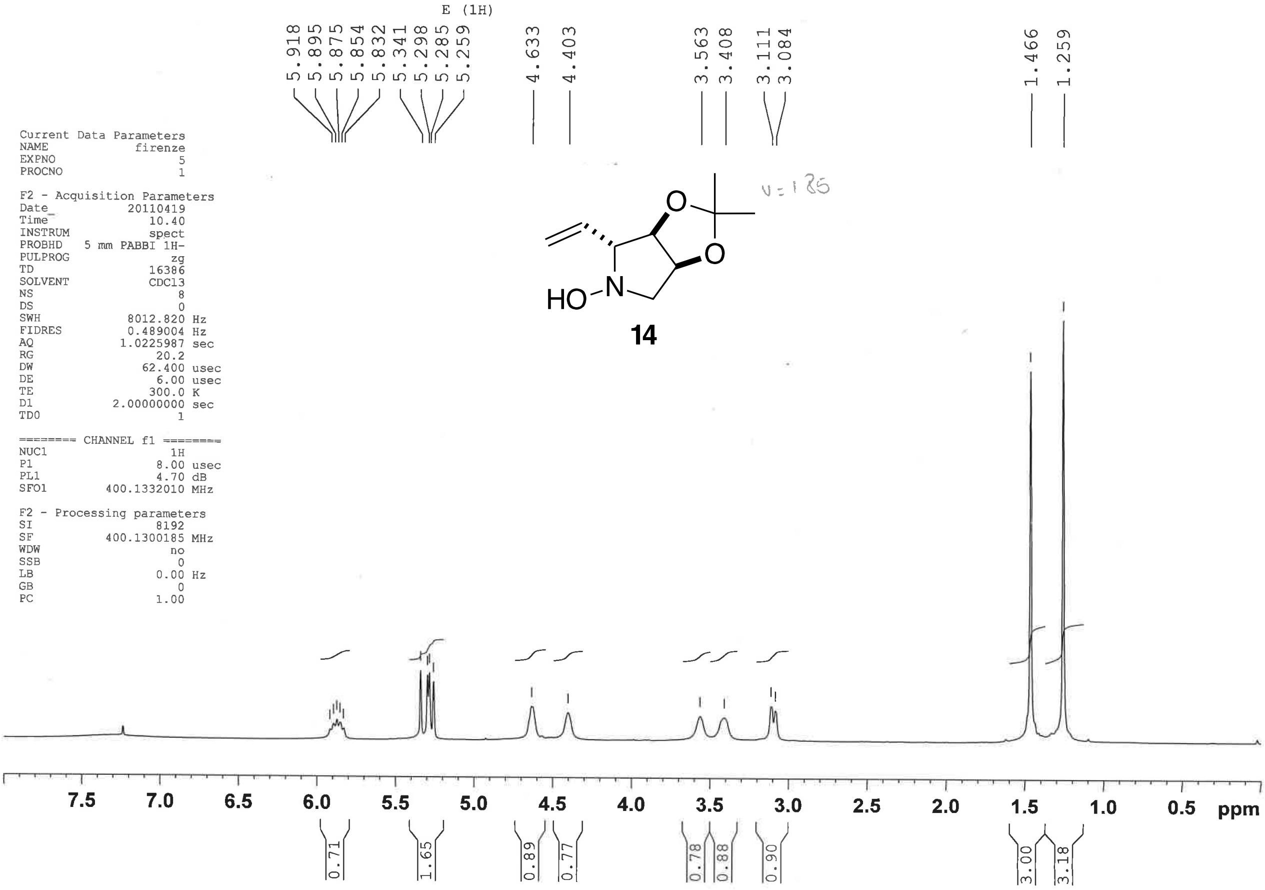

Print of window 80: MS Spectrum

Acq. Operator : ELENA

Location : Vial 1

Injection Date : 20-Jun-08, 09:59:18
Acq. Method : TEST.M
Analysis Method : C:\CHEM32\1\METHODS\TEST.M
Last changed : 6/20/2008 9:58:18 AM by MADDA
(modified after loading)

MS Spectrum

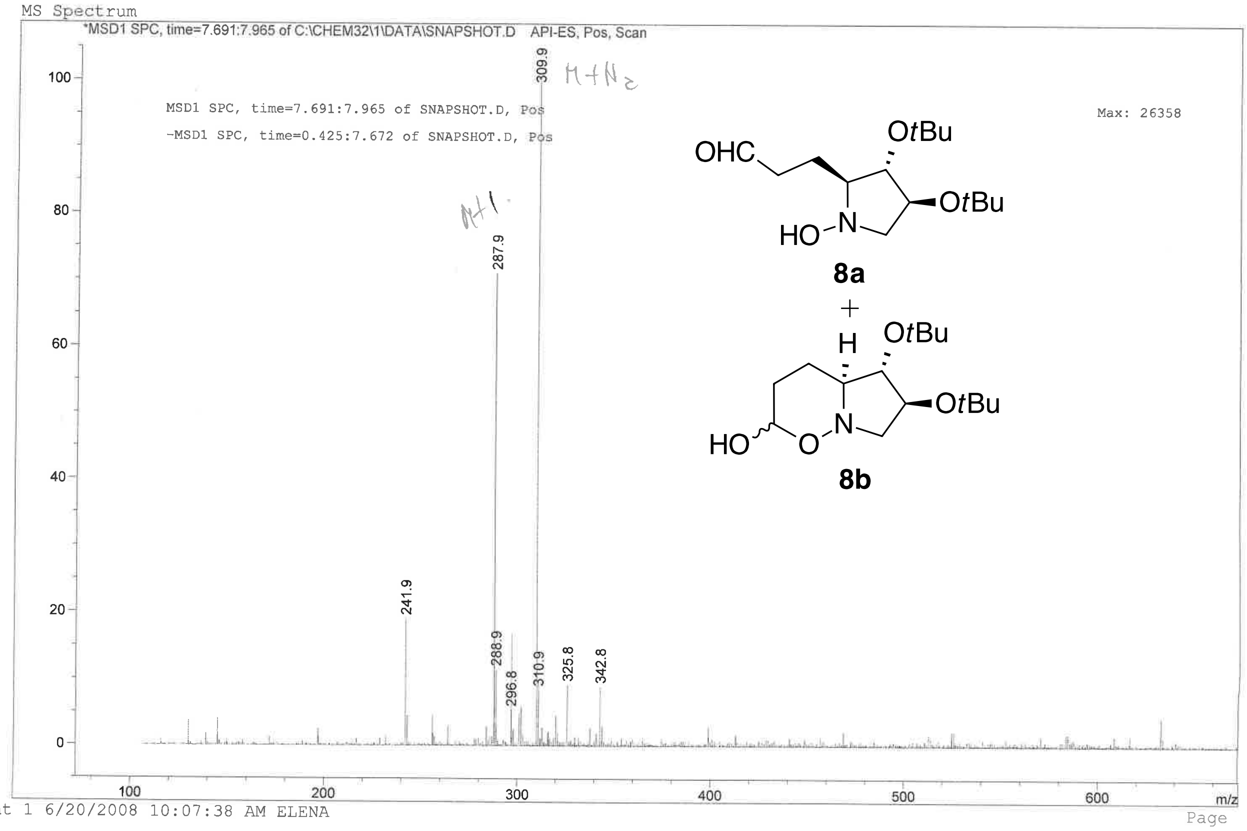

Instrument 1 6/20/2008 10:07:38 AM ELENA

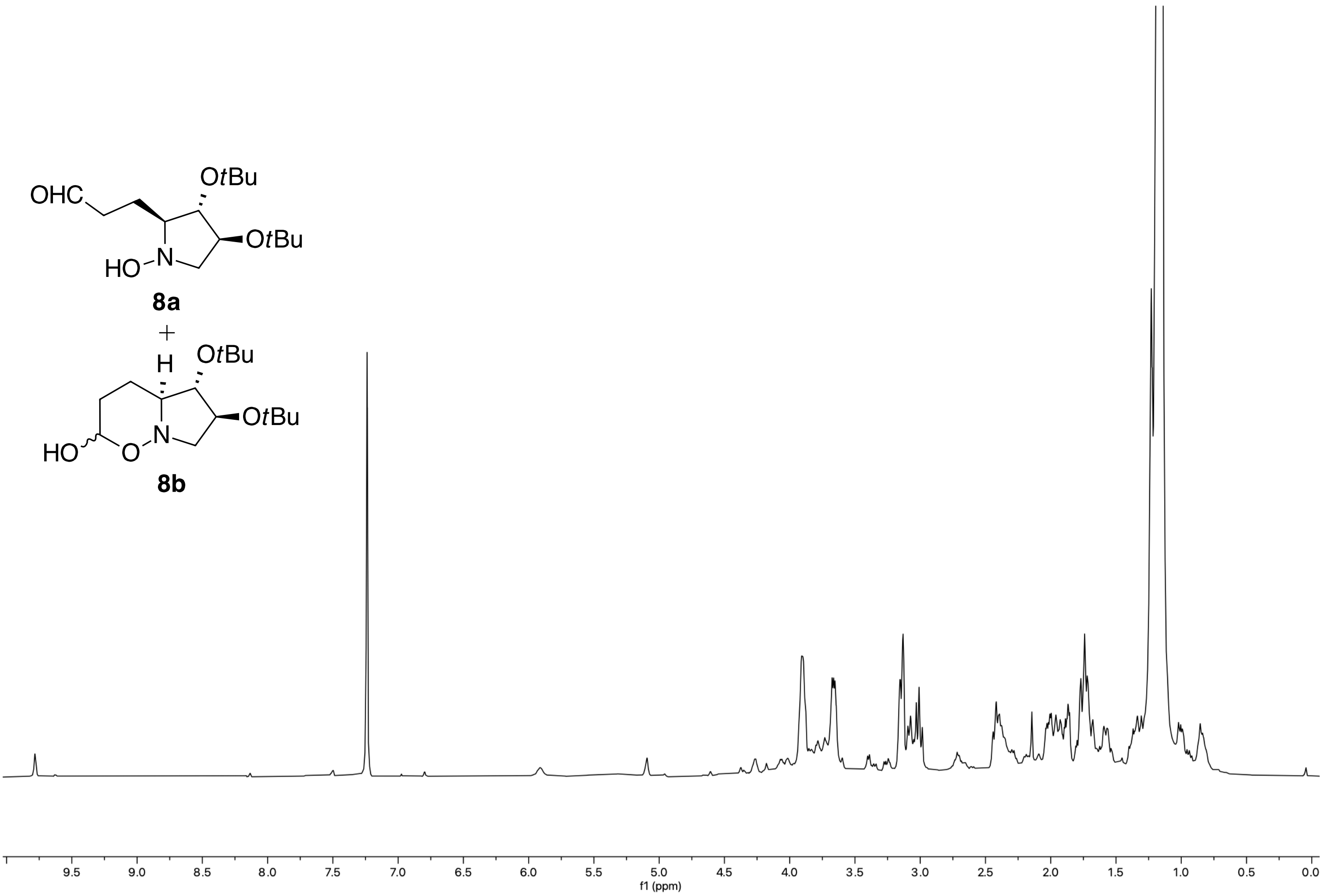
OHC
OtBu
OtBu
HO
N
H
OtBu
OtBu
HO
O
N
9.5 9.0 8.5 8.0 7.5 7.0 6.5 6.0 5.5 5.0 4.5 4.0 3.5 3.0 2.5 2.0 1.5 1.0 0.5 0.0
f1 (ppm)

8a + **8b**

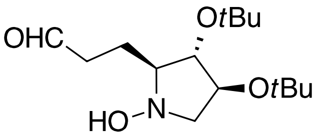
OHC
OtBu
OtBu
HO
N

8a

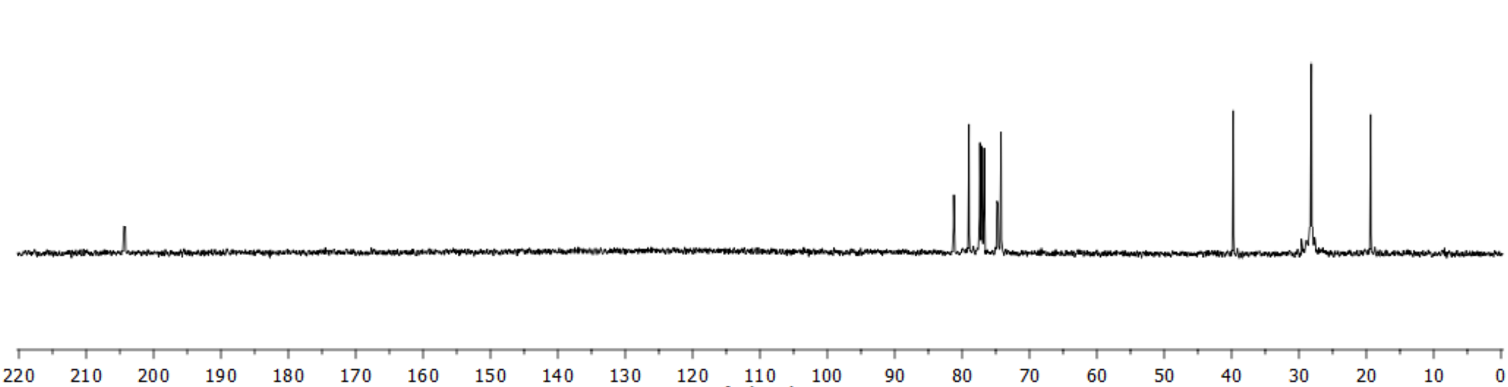
220 210 200 190 180 170 160 150 140 130 120 110 100 90 80 70 60 50 40 30 20 10 0

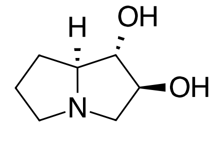
H
OH
OH
N

3

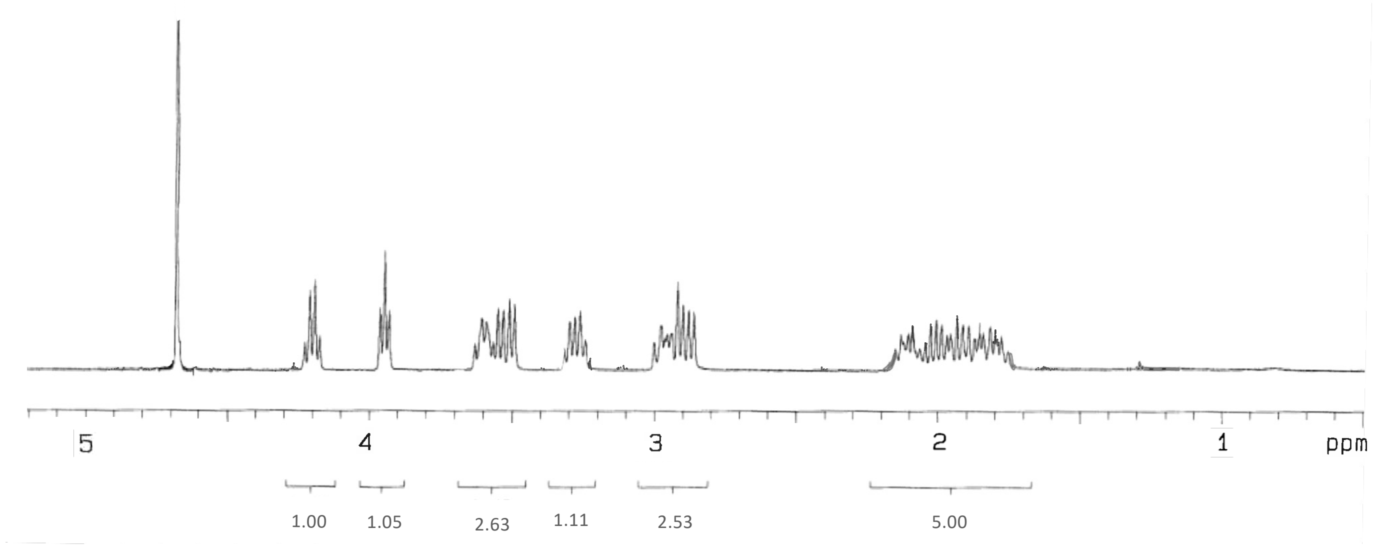
5
4
3
2
1
ppm
1.00
1.05
2.63
1.11
2.53
5.00

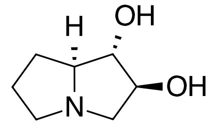
H
OH
OH
N

3

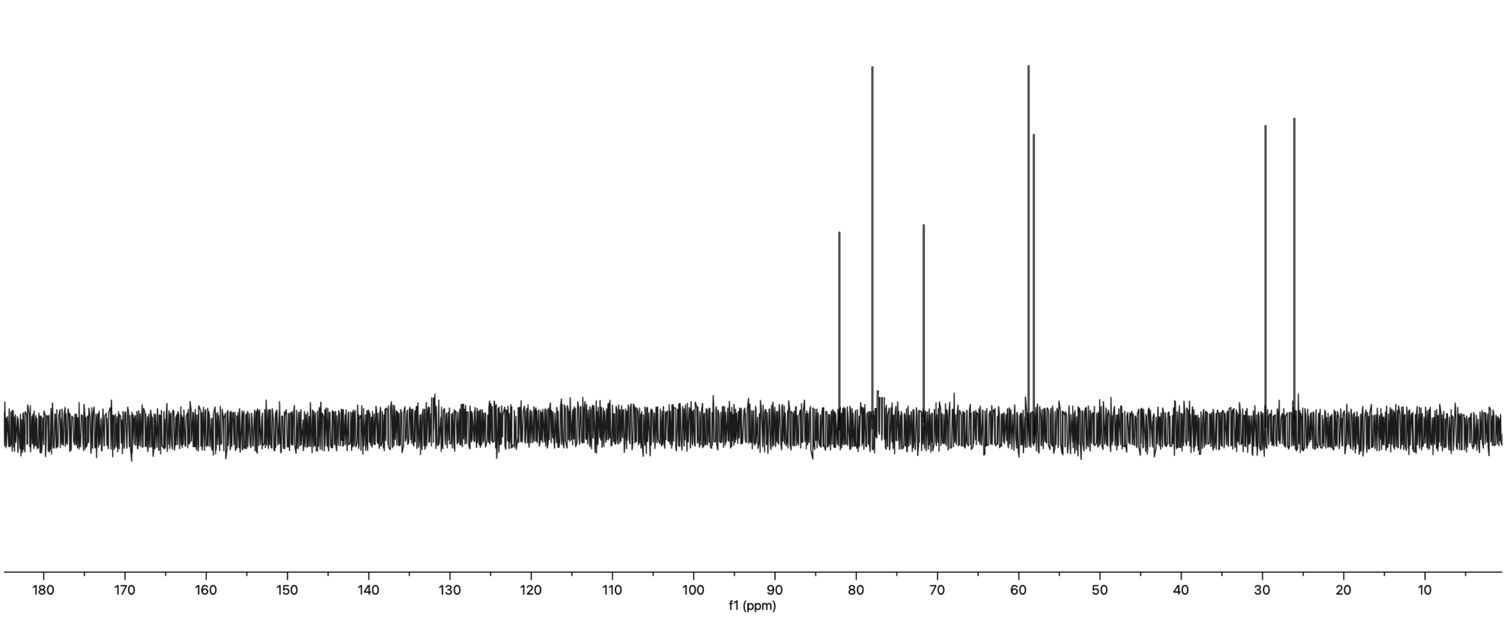
82.0977
77.8895
71.7463
58.8970
58.0321
29.6748
26.2733
180
170
160
150
140
130
120
110
100
90
80
70
60
50
40
30
20
10
f1 (ppm)

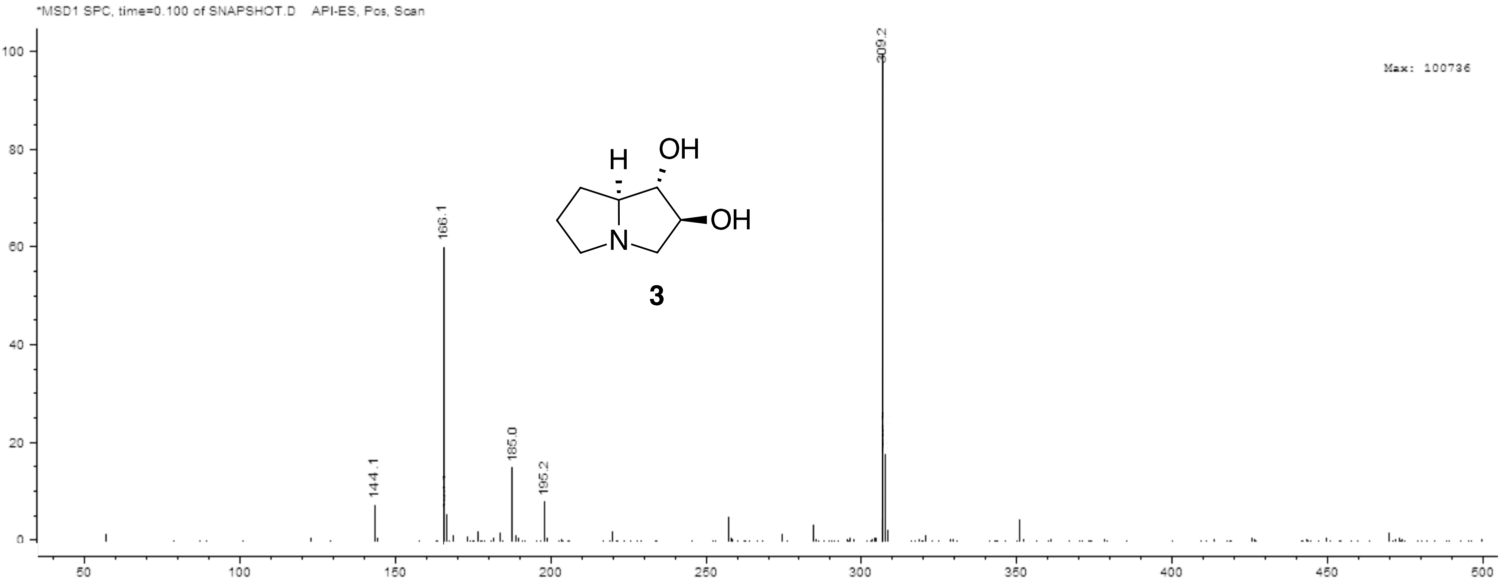
*MSD1 SPC, time=0.100 of SNAPSHOT.D API-ES, Pos, Scan
Max: 100736
309.2
166.1
185.0
195.2
144.1
3

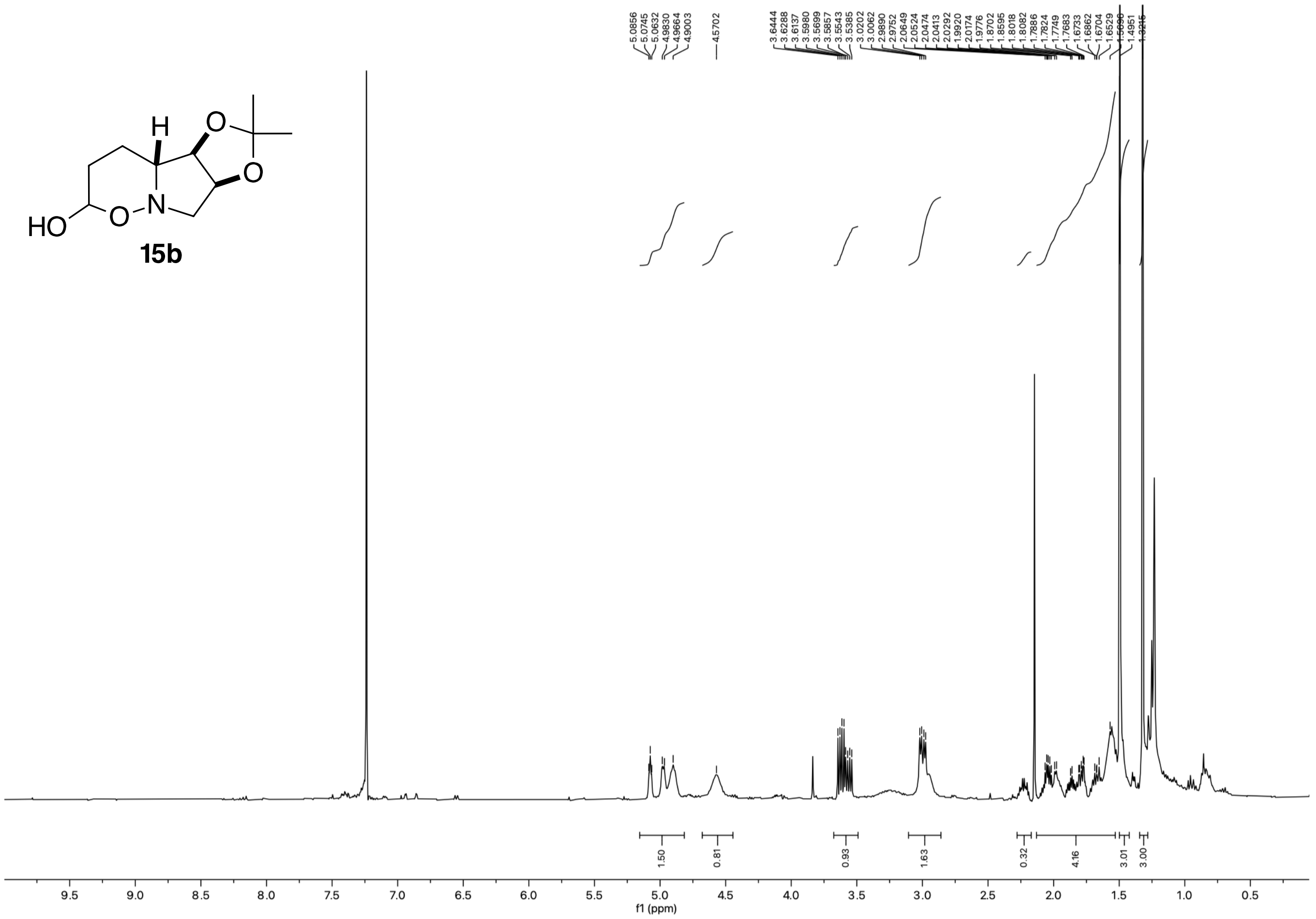
15b
f1 (ppm)

Print of window 80: Spectrum

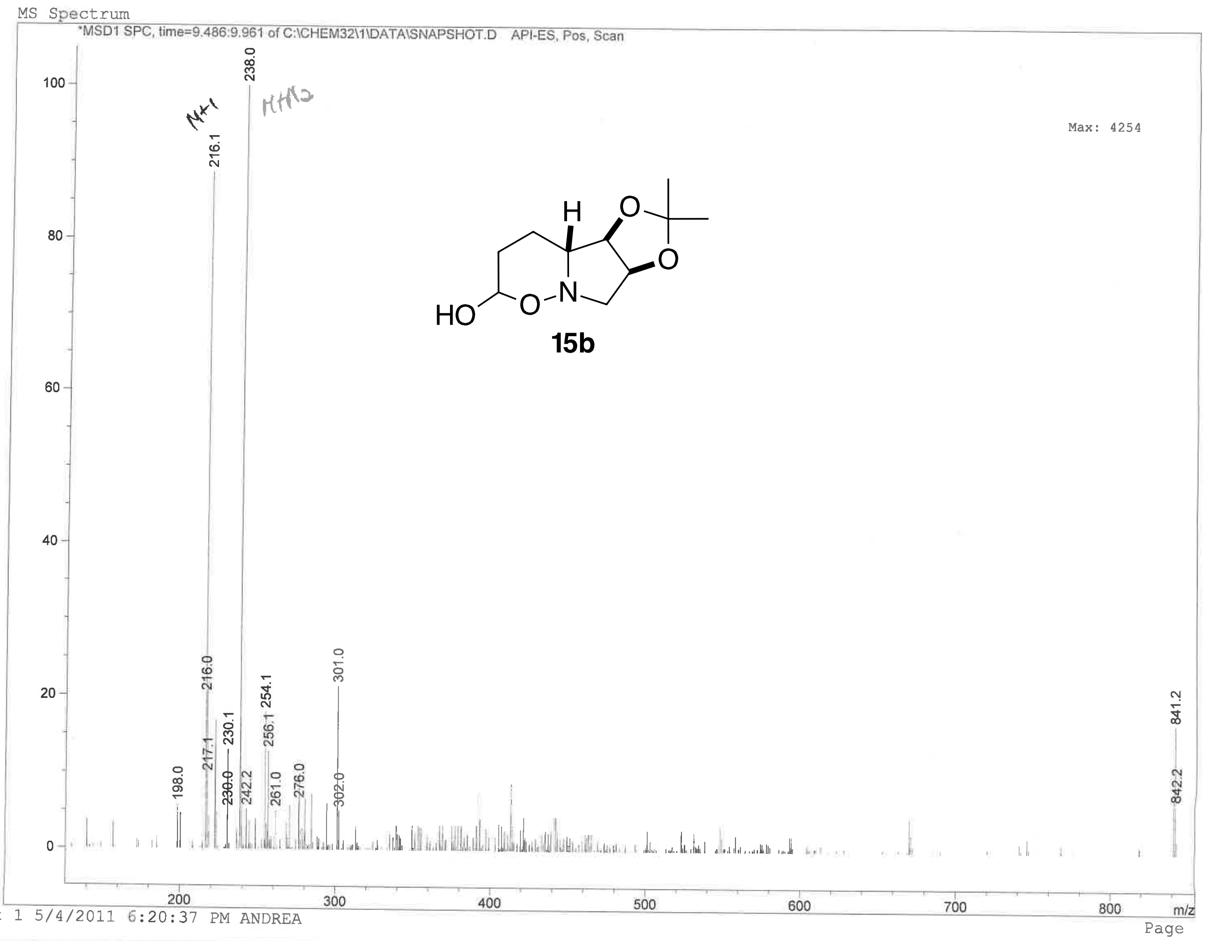

Instrument 1 5/4/2011 6:20:37 PM ANDREA

Page 1 of 1

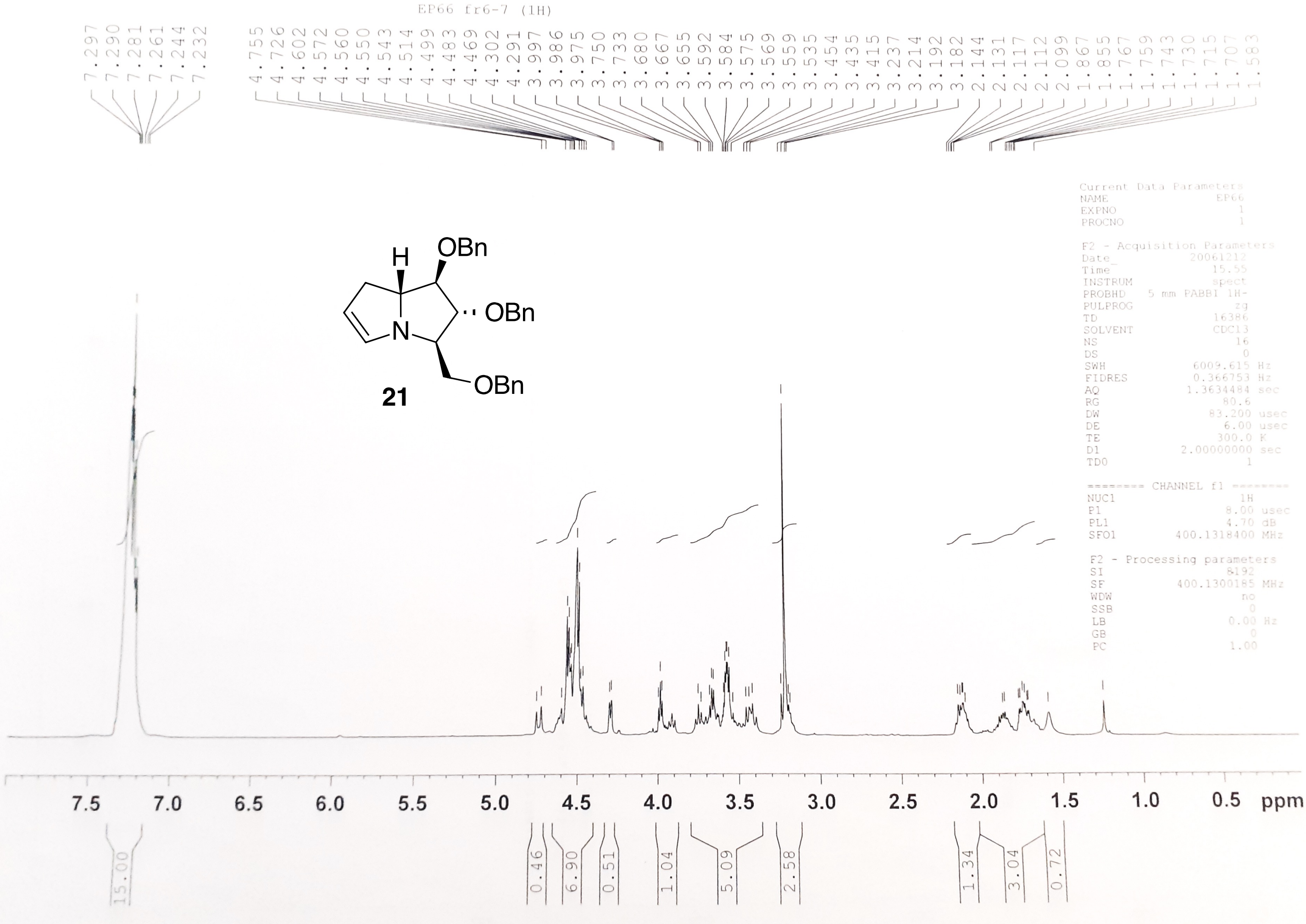

Print of window 80: MS Spectrum

Acq. Operator : alex

Location : Vial 1

Injection Date : 21-Dec-06, 11:20:24
Acq. Method : ACN.M
Analysis Method : C:\CHEM32\1\METHODS\ACN.M
Last changed : 12/21/2006 11:12:58 AM by ale
(modified after loading)

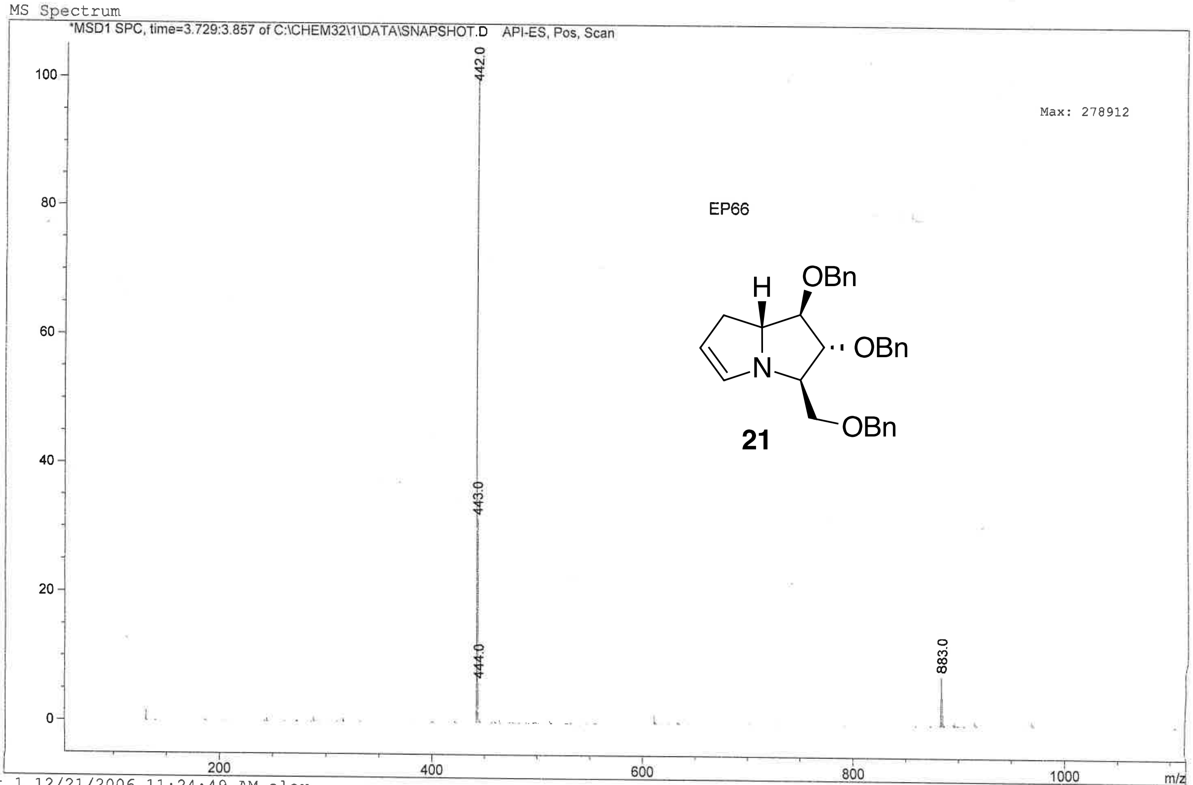

Instrument 1 12/21/2006 11:24:49 AM alex

Page 1 of 1

Print of window 80: MS Spectrum

Acq. Operator : E LENA
Acq. Instrument : Instrument 1
Injection Date : 4/15/2008 6:26:27 PM
Method : C:\CHEM32\1\METHODS\SIOTT .M
Last changed : 4/15/2008 6:04:27 PM by BEA
(modified after loading)

Location : -
Inj : 1

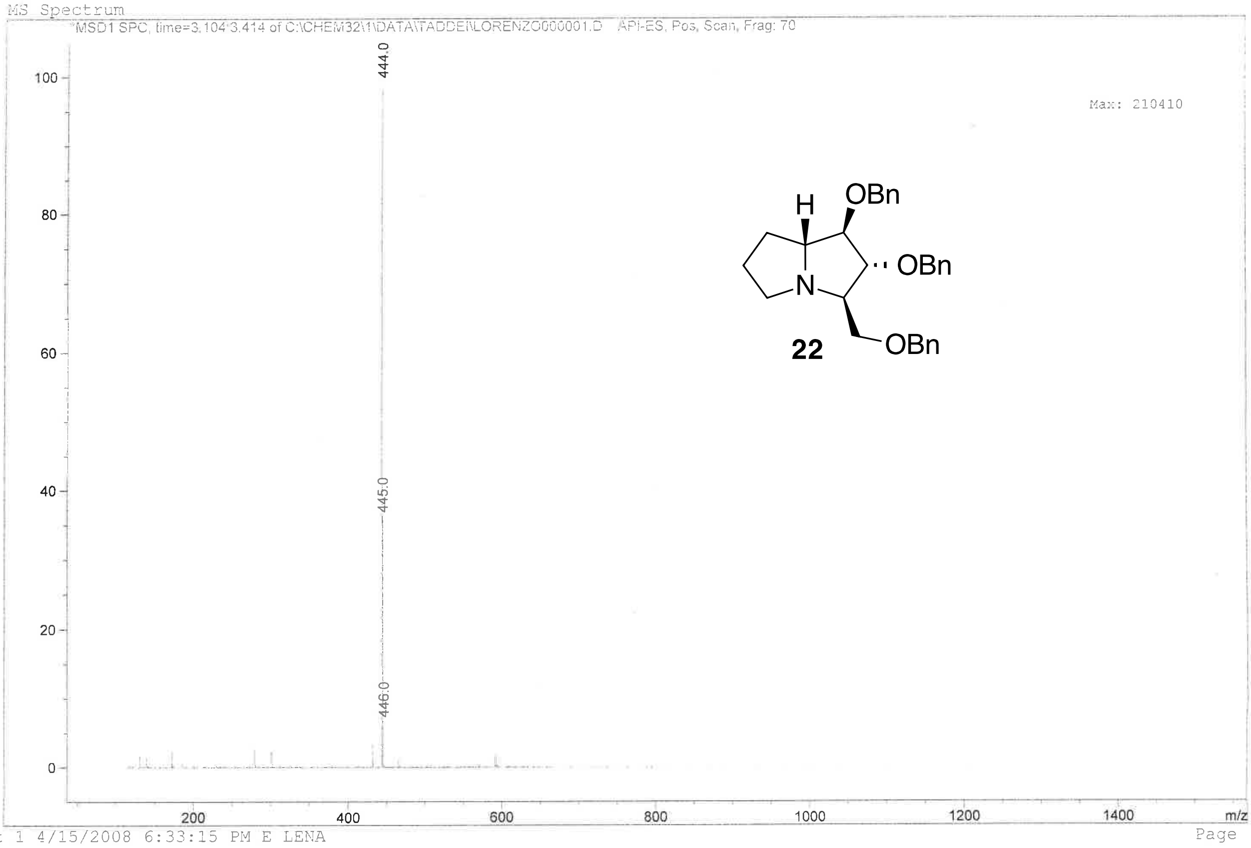

Instrument 1 4/15/2008 6:33:15 PM E LENA

Page 1 of 1

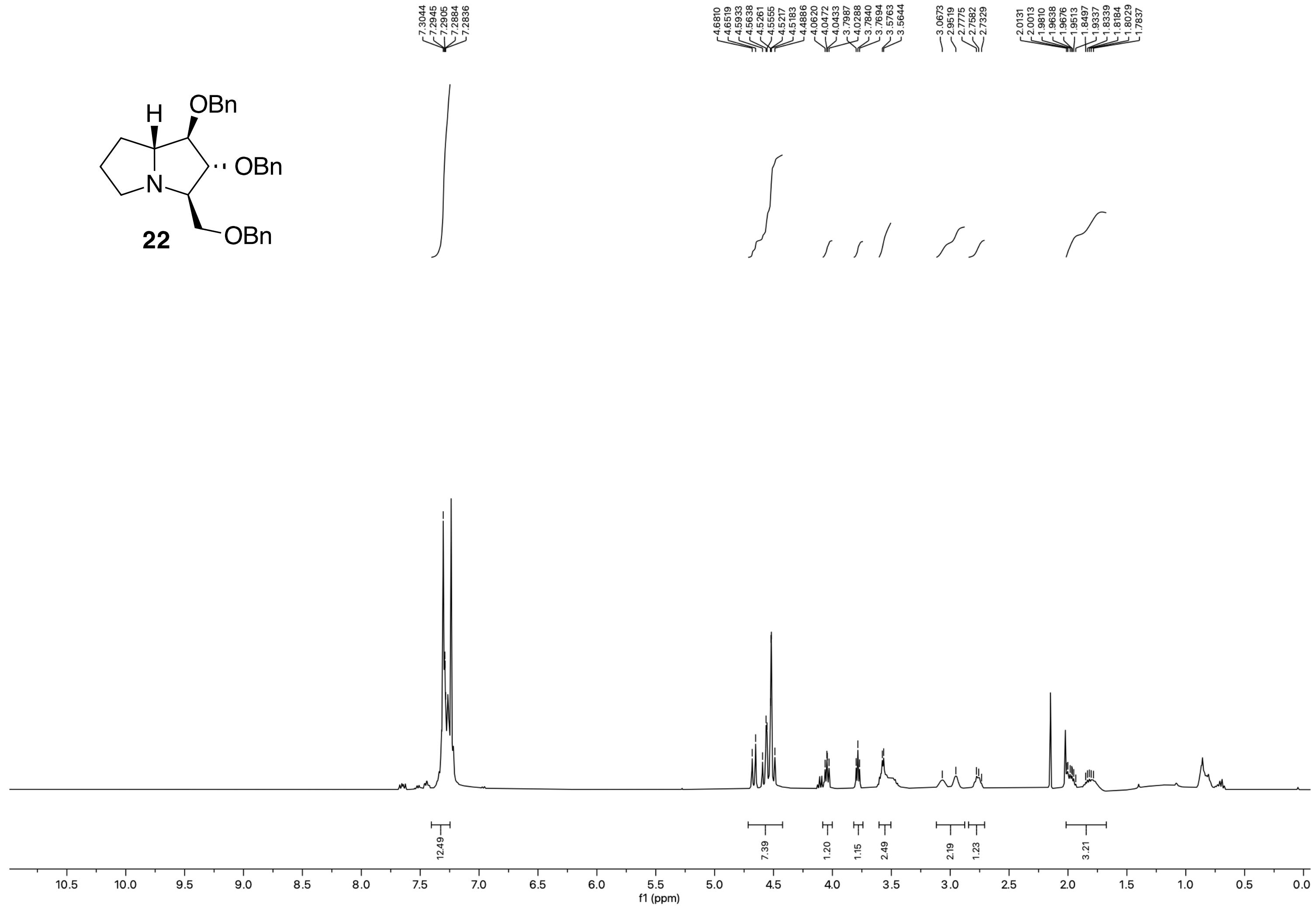
7.3044
7.2945
7.2905
7.2884
7.2836
4.6810
4.6519
4.5933
4.5638
4.5555
4.5261
4.5217
4.5183
4.4886
4.0620
4.0472
4.0433
4.0288
3.7987
3.7840
3.7694
3.5763
3.5644
3.0673
2.9519
2.7775
2.7582
2.7329
2.0131
2.0013
1.9810
1.9638
1.9676
1.9513
1.8497
1.9337
1.8339
1.8184
1.8029
1.7837
H
OBn
OBn
N
OBn
22
12.49
7.39
1.20
1.15
2.49
2.19
1.23
3.21
f1 (ppm)

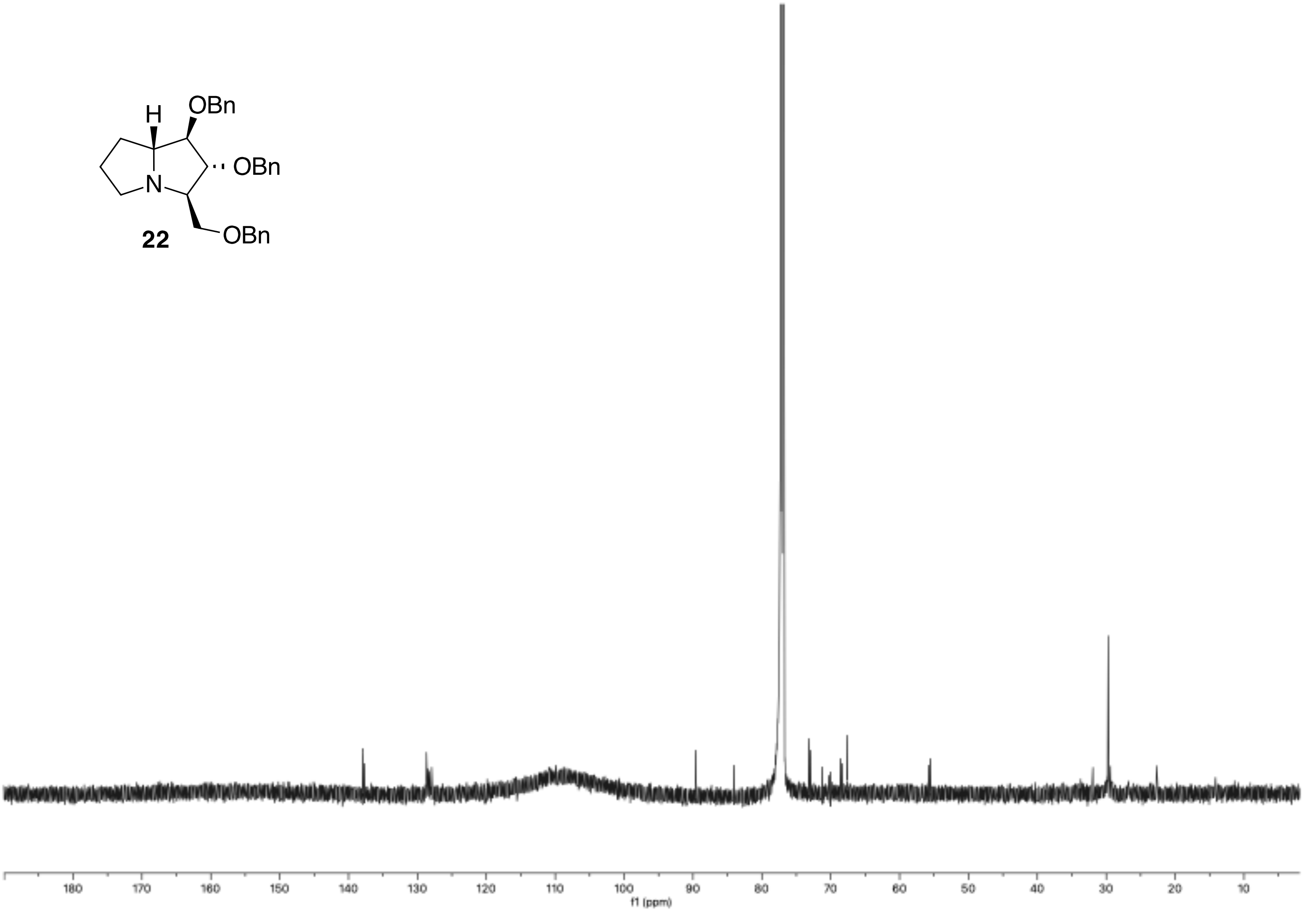
H
OBn
OBn
N
OBn
22
f1 (ppm)
